# Effect of noninvasive ventilation on 6-month mortality in patients with acute cardiogenic pulmonary oedema: a retrospective study dedicated to older persons

**DOI:** 10.1007/s11739-025-04012-3

**Published:** 2025-06-15

**Authors:** Linda Haddi, Laure Valerio, Pascal Bilbault, Georges Kaltenbach, Elise Schmitt

**Affiliations:** 1 Geriatric Department, University Hospital of Strasbourg, Strasbourg, France; 2 Public Health Department, Hospital of Dracenie, Draguignan, France; 3 Emergency Department, University Hospital of Strasbourg, Strasbourg, France; 4 UR-3072, University of Strasbourg, Strasbourg, France

**Keywords:** Acute cardiogenic pulmonary oedema, Noninvasive ventilation, Older person, Mortality

## Abstract

Noninvasive ventilation (NIV) effectively treats acute cardiogenic pulmonary oedema (ACPE), leading to quicker clinical improvement and reduced intubation needs than medical treatment alone. However, the impact of NIV on mortality in patients with ACPE is unclear. The primary objective was to evaluate the impact of NIV on 6-month mortality in older patients with ACPE compared with medical treatment. Secondary objectives included assessing the effects of NIV on length of hospital stay and rehospitalisation. This retrospective study included patients aged ≥ 75 years with ACPE (acute respiratory distress and/or RR ≥ 25 cycles/min and/or paCO2 ≥ 45 mmHg and/or pH < 7.35 and/or uncorrected hypoxemia). Exclusion criteria: positive for SARS-CoV-2 and contraindication to NIV. Of the 186 patients admitted to emergency care with ACPE and eligible for NIV, 104 received NIV and 82 received medical treatment. Survival analyses were performed using a multivariate Cox model and adjusting for confounding factors. NIV was not significantly linked to reduced mortality risk (HR = 0.82, *p* = 0.51), except for in the acidosis subgroup (HR = 0.24, *p* = 0.01). No difference was observed in the length of hospital stay or in terms of rehospitalisation. NIV in older patients with ACPE did not significantly decrease 6-month mortality, except in the subgroup with respiratory acidosis, when the risk of death was reduced by 75%. The use of NIV in older patients with ACPE should be limited to patients with acidosis, to see their risk of death significantly reduced. *Trial registration*: The study protocol has been retrospectively registered on ClinicalTrials.gov (NCT06107257, 2023-10-30).

## Introduction

The aging of the global population has significant societal implications. Geriatrics addresses the care of frail, multimorbid older persons to maintain their prior autonomy amid the inevitable decompensation of chronic pathologies. Considering therapeutic intensity and optimal intrahospital orientation is vital for aligning care objectives and patient functional reserve.

Chronic heart failure becomes increasingly prevalent with age, affecting over 10% of individuals aged 70 years and older. This condition is associated with a high annual mortality rate ranging from 16 to 20% in older persons, which increases further with age [1].

Acute heart failure (AHF) may present as acute cardiogenic pulmonary oedema (CPE), a life-threatening condition requiring urgent management. Currently, CPE is the second most common indication for noninvasive ventilation (NIV) [2] and among the earliest established uses of this intervention [3].

NIV is a widely used therapy acknowledged for its ability to improve respiratory symptoms and reduce the need for intubation [4–6]. It is often preferred over standard treatment because it offers a more effective alternative, especially in highly dependent or fragile patients for whom invasive mechanical ventilation may be inappropriate. NIV should be administered alongside optimal medical therapy without delaying acute coronary syndrome management.

Current guidelines recommend initiating NIV based on clinical signs of respiratory distress (e.g., RR > 25/min, SpO₂ < 90% despite supplemental oxygen) and/or hypercapnia (PaCO₂ > 45 mmHg), using either continuous positive airway pressure (CPAP) or bilevel positive airway pressure (BiPAP) modes [7–12].

In patients with AHF, NIV may also provide hemodynamic benefits through the application of positive end-expiratory pressure (PEEP). This increases transpulmonary pressure, leading to higher pulmonary vascular resistance, reduced venous return, and notably, decreased left ventricular afterload, which may enhance left ventricular systolic volume [13].

However, the impact of NIV on mortality in older patients, patients with cardiac failure, and polymorbid patients remains inconclusive, as survival alone may not be the primary therapeutic objective. We, therefore, conducted a retrospective single-center study to compare the effectiveness of NIV combined with standard therapy versus standard therapy alone in older patients presenting with CPE in the emergency department. The primary outcome was 6-month mortality. Secondary outcomes included improvement in respiratory distress, need for endotracheal intubation and/or mechanical ventilation, hospital length of stay, rehospitalisation, and impact on blood gases values.

## Methods

### Study design

This was an observational, retrospective, longitudinal, single-center study using survival analysis. Patient inclusions occurred between January and October 2021 in the Adult Emergency Department of the University Hospital of Strasbourg (UHS), France.

### Study population

Older patients admitted to the emergency department at the UHS with a diagnosis of CPE.

A. Inclusion criteria
A Age 75 years and above
B Admission to the Emergency Department between January 1 and October 31, 2021
C Diagnosis of cardiogenic pulmonary oedema
D Indication for non-invasive ventilation (NIV) according to current recommendations (2006 consensus criteria and guidelines from the European Society of Cardiology (ESC)).
B. Exclusion criteria
E Absolute contraindication to NIV
F Non-cardiogenic causes of respiratory distress
G Positive SARS-CoV-2 RT-PCR result upon admission

### Data collection

Two patient groups were defined: the first underwent management with NIV, while the second received standard therapy alone.

Patients older than 75 years were identified using two strategies: (1) Manual review of the paper emergency register, which documented admissions to the acute care zone designated for cases with hemodynamic instability or NIV indications; (2) Search via computerized coding of principal and/or associated diagnoses at discharge from the emergency consultation.

Comprehensive data were extracted from electronic medical records (Dxcare^®^, Maincare Solutions), including demographics, comorbidities, clinical presentation, biological data, ventilatory parameters, imaging, and treatment details. Functional status was assessed using the Groupes Iso-Ressources (GIR) score. Additional information included post-emergency referral, hospital length of stay, and in-hospital mortality. For patients without 6-month follow-up data in the hospital record system, survival status was obtained from general practitioners, nursing home coordinators, or the French national death registry.

The Charlson Comorbidity Index was calculated for all patients. Data were compiled using Excel^®^ (Microsoft Corporation, Redmond, WA, USA).

### Statistical analysis

Statistical analysis was performed using R software (R Development Core Team, 2008). Quantitative variables are presented as the mean ± standard deviation, and qualitative variables as counts and percentages. Univariate analysis was performed using Student's t test for equal variances, the Mann‒Whitney test for unequal variances, and the chi-square test for categorical variables.

Survival curves were generated using Kaplan‒Meier analysis and assessed by the log-rank test. A multivariate Cox proportional hazards regression model was used to identify variables independently associated with 6-month mortality.

The initial variable selection included the most significant frailty variable among those with dementia, GIR score, and Charlson Comorbidity Index score through a preliminary analysis. Additionally, variables included in the model were those with a significant univariate association with mortality or clinical relevance: sex, age, pH, paO2, and smoking status.

A subgroup analysis focusing on pH and capnia was performed.

The results are expressed as hazard ratios (HRs) and 95% confidence intervals. A *p* value < 0.05 was considered to indicate statistical significance.

## Results

### Baseline characteristics

Using the paper emergency register, and excluding those under 75 years of age, each computerized record of the 3500 outpatients managed from January to October 2021 was screened. Ninety-five patients were included in the NIV + group (standard treatment + NIV), while 5 were included in the NIV– group (standard treatment only).

Through computer coding of principal and/or associated diagnoses at the end of their emergency consultation, 2050 patients aged 75 and over in 2021 were identified with specific heart failure codes. Of these, 86 patients were managed over the same period, including 9 in the NIV + group and 77 in the NIV- group.

**Fig. 1:**
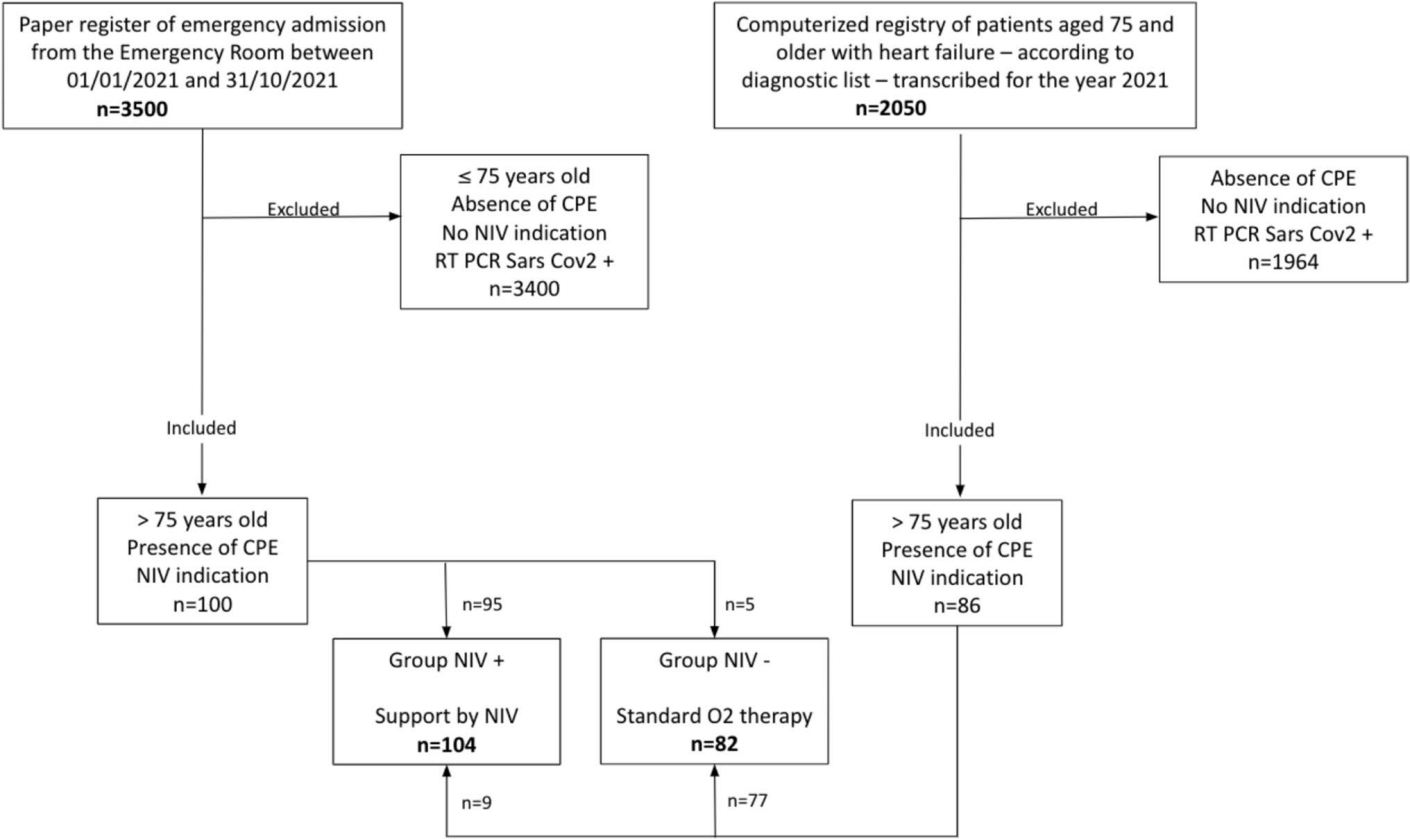
Flow chart. *NIV* + Noninvasive Ventilation group, *NIV*– group without Noninvasive Ventilation, *CPE* cardiogenic acute pulmonary oedema

A total of 186 patients over 75 years of age were admitted to the emergency department with CPE, all of whom were eligible for NIV. Among them, 104 patients (56%) received NIV (NIV + group), and 82 (44%) received standard medical treatment without NIV (NIV– group) (Fig. 1).

The mean age of the study population was 86 ± 6 years, ranging from 75 to 103 years, with 53.8% (*n* = 100) aged 85 years and older. There was no significant difference in age between the NIV + and NIV– groups (85 ± 6 vs. 87 ± 7 years, *p* = 0.07). Women represented the majority of the study cohort (64%), with a mean body mass index (BMI) of 27 kg/m^2^. Tobacco use was twice as frequent in the NIV + group compared to the NIV– group (40% vs. 20%, *p* = 0.003).

The average Charlson Comorbidity Index was 8.7 ± 2.4, ranging from 4 to 16, and did not significantly differ between the two groups (8.7 ± 2.1 for NIV + vs. 8.9 ± 2.3 for NIV–, *p* = 0.22). Over half of the population had impaired functional autonomy (GIR score < 4), with 4.5% missing data. The NIV + group exhibited lower autonomy levels (70% vs. 49%, *p* = 0.003). Major neurocognitive disorders were also more prevalent in the NIV + group (48% vs. 35%, *p* = 0.006), though underreporting in the NIV + group may have affected this result (missing data: 12.5% vs. 3.7%)(Table 1).

**Table 1:** Comparison of the baseline characteristics between NIV + group and NIV– group

Characteristics	NIV + *N* = 104/186	NIV– *N* = 82/186	*p* value
Variable			
Sex ratio M/F	0.38	0.33	0.490
Age at baseline, y (Mean (SD))	85.4 (6.1)	87.1 (6.6)	0.435
Medical history			
Charlson Comorbidity Index score 0–10 (Mean (SD))	8.7 (2.1)	8.9(2.3)	0.220
Chronic obstructive pulmonary disease (Ratio)	0.22	0.15	0.195
History of tobacco (Ratio)	0.40	0.20	0.003
BMI, kg/m^2^ (Mean (SD))	27.5 (6.6)	27.5 (7.0)	> 0.9
Cognitive disorders (Ratio)	0.48	0.35	0.006
GIR score > 4 (Ratio)	0.30	0.51	0.003
Heart failure (Ratio)	0.90	0.96	0.114
Vital parameters			
Systolic blood pressure, mmHg (Mean (SD))	152.7 (38.8)	139.3 (27.3)	< 0.010
Diastolic blood pressure, mmHg (Mean (SD))	78.7 (21.4)	79.1 (16.3)	0.870
Heart rate, beats per min (Mean (SD))	90.8 (26.4)	88.5 (26.6)	0.550
Respiratory rate, breaths per min (Mean (SD))	31.8 (7.9)	29.2 (5.7)	0.150
Oxygen saturation, % (Mean (SD))	79.0 (11.2)	83.5 (8.52)	0.015
Gasometrics parameters			
Arterial pH (Mean (SD))	7.3 (0.1)	7.4 (0.1)	< 0.001
Arterial partial pressure of O2 with oxygen, mmHg (Mean (SD))	116.7 (81.3)	105.9 (59.9)	0.680
Arterial partial pressure of CO2 with oxygen, mmHg (Mean (SD))	52.9 (19.1)	45.1 (10.8)	0.001
Bicarbonate, mmol/l (Mean (SD))	26.3 (6.2)	27.5 (5.3)	0.160
Lactates, mmol/l (Mean (SD))	1.8 (1.6)	1.2 (0.7)	0.140
Relation of PaO2 and inspired O2 (PaO2/FiO2) (Mean (SD)	279.1 (157.7)	284.5 (121.7)	0.110
pH < 7.35 (Ratio)	0.58	0.15	< 0.001

*y* year, *N* number, *BMI* body mass index, *SD* standard deviation, *NIV* + noninvasive ventilation group, *NIV*– group without noninvasive ventilation, *GIR* group Iso Resources, *O2* oxygen, *CO2* carbon dioxide, *paO2* arterial partial pressure of O2, *paCO2* arterial partial pressure of CO2, *FiO2* fraction of inspired O2

The NIV + group showed lower systolic blood pressure (152.7 ± 38.8 mmHg vs. 139.3 ± 27.3 mmHg, *p* < 0.01) and lower oxygen saturation (79.0 ± 11.2% vs. 83.5 ± 8.5%, *p* = 0.015). Gasometric analysis revealed that the NIV + group had more pronounced respiratory acidosis (mean pH 7.3 ± 0.1 vs. 7.4 ± 0.1, *p* < 0.001) and greater hypercapnia (PaCO₂ 52.8 ± 19.1 mmHg vs. 45.1 ± 10.8 mmHg, *p* = 0.001).

### Primary outcome: impact of NIV on mortality

#### Univariate analyses

Univariate comparisons revealed no significant differences in survival between the NIV + and NIV– groups at any of the measured timepoints: day 2 (4% vs. 1%, *p* = 0.39), day 7 (5% vs. 6%, *p* = 0.75), day 14 (6% vs. 11%, *p* = 0.2), day 30 (12% vs. 15%, *p* = 0.53), month 3 (18% vs. 24%, *p* = 0.31), or month 6 (26% vs. 32%, *p* = 0.39) (Table 2).

**Table 2:** Population mortality

	NIV + *N* = 104/186	NIV– *N* = 82/186	*p* value
Mortality			
Day 2, %	4	1	0.39
Day 7, %	5	6	0.75
Day 14, %	6	11	0.20
Day 30, %	12	15	0.53
Month 3, %	18	24	0.31
Month 6, %	26	32	0.39

*NIV* + noninvasive ventilation group, *NIV*– group without noninvasive ventilation

**Fig. 2:**
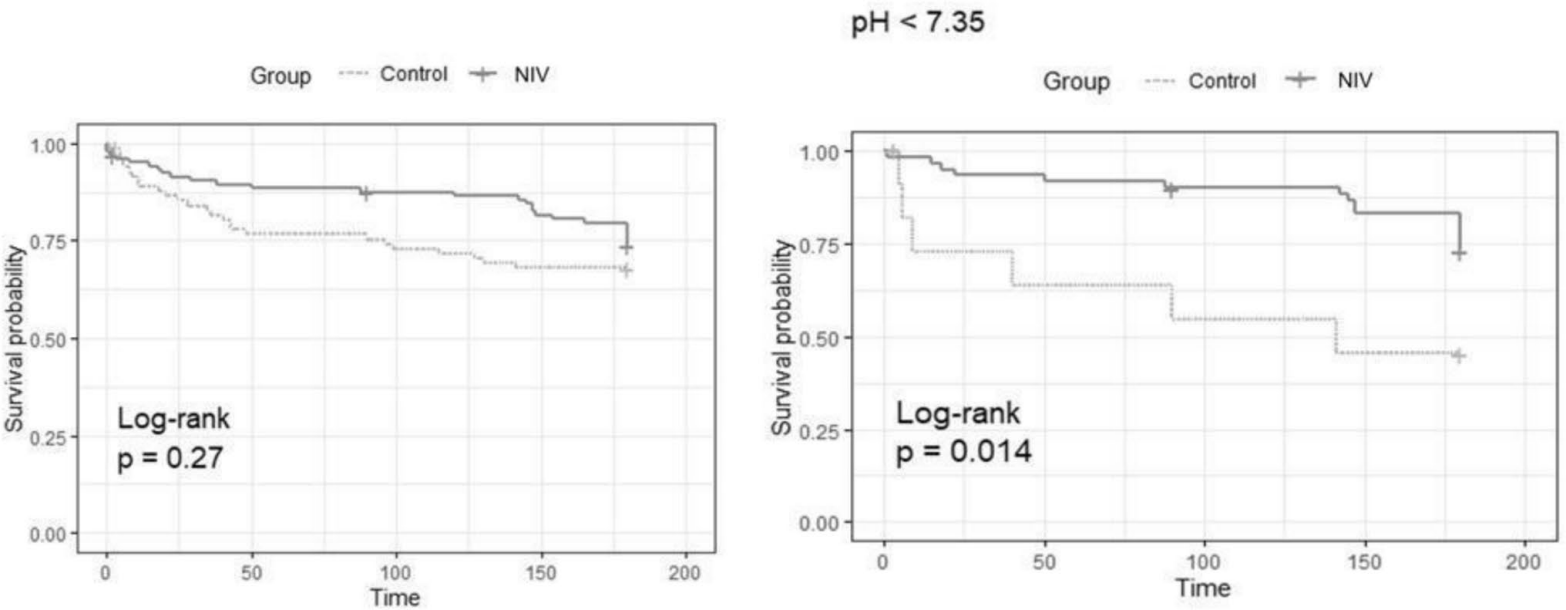
Kaplan–Meier Analysis: Cumulative 180 days survival in the two groups for the global population (left) and in the population with respiratory acidosis (right). Group Control, NIV– group; NIV, NIV + group

The log rank test confirmed no statistically significant difference in cumulative 180-day survival between the two groups (*p* = 0.274) (Fig. 2).

#### Multivariate analysis

To assess the independent effect of NIV on mortality, we used multivariate Cox proportional hazards modeling, to adjust for confounding factors (variables either significantly associated with increased mortality or treatment group in univariate analysis or considered clinically relevant) such as age, sex, Charlson Comorbidity Index, smoking status, pH, paO2.

Overall, NIV did not exhibit a significant association with a reduced risk of death in the entire cohort (HR 0.76, *p* = 0.40). However, older age (HR = 1.10 [95% CI: 1.05–1.15], *p* < 0.001) and higher Charlson Comorbidity Index (HR = 1.16 [1.02–1.31], *p* = 0.022) were independently associated with increased mortality.

In patients with respiratory acidosis (pH < 7.35), the use of NIV was associated with a significant 76% reduction in mortality risk (HR = 0.24 [0.08–0.72], *p* = 0.01). (Table 3 and Fig. 2).

**Table 3:** Impact of NIV on mortality in patients with hypercapnia, respiratory acidosis and without respiratory acidosis

	Hazard ratio (IC 95%)	*p* value
NIV +, sub-group paCO2 > 45 mmHg	0.70 [0.23; 2.11]	0.52
*N* = 98 *E* = 28		
NIV +, sub-group pH < 7.35	0.24 [0.08; 0.72]	0.01
*N* = 74 *E* = 22		
Sub-group pH > 7.35	0.92 [0.44; 1.94]	0.83
*N* = 114 *E* = 31		

*NIV* + noninvasive ventilation group, *NIV*– group without noninvasive ventilation, *paCO2* arterial partial pressure of CO2, *E* Event

### Secondary outcomes

#### Length of hospital stay

There was no significant difference in the length of hospital stay between the groups (13 ± 14 days for the NIV + group vs. 11 ± 6 days for the NIV– group, *p* = 0.76).

#### Six months rehospitalisation

The mean number of readmissions for CPE within six months was low in both groups (0.4 ± 0.8 versus 0.3 ± 0.5, *p* = 0.32).

Overall, fifty-two percent of patients in the NIV + group were readmitted to the hospital within 6 months versus forty-two percent in the NIV- group (*p* = 0.179).

#### Blood pH

The NIV + group exhibited a significantly greater improvement in arterial pH following emergency care compared to the NIV– group (+ 0.07 vs. + 0.03, *p* = 0.023).

#### Predictive factors for NIV prescription

Patients in the NIV + group had significantly lower oxygen saturation (79.0 ± 11.2% vs. 83.5 ± 8.5%, *p* = 0.015) and lower arterial pH (7.3 ± 0.1 vs. 7.4 ± 0.1, *p* < 0.001), as well as higher PaCO₂ values (52.9 ± 19.1 mmHg vs. 45.1 ± 10.8 mmHg, *p* = 0.001). Additionally, patients in the NIV + group had lower functional autonomy (lower GIR score) and a higher prevalence of cognitive impairment. Pulmonary infection was also more frequent in the NIV- group (20% vs. 12%, *p* = 0.027).

#### Post-emergency department referral

Thirty-four patients (33%) in the NIV group versus 5 patients (6.1%) in the control group (*p* < 0.0001) were admitted to a critical care unit (Intensive Care Unit/Continuous Internal Medicine Care and Medical Resuscitation). The remaining patients were admitted to conventional medical wards (Geriatrics, Internal Medicine, Cardiology, Respiratory medicine, Nephrology), with 70 patients (67%) in the NIV + group and 77 patients (94%) in the NIV- group (*p* < 0.0001).

## Discussion

### Primary outcome

Over a 10-month period, 186 patients aged 75 years or older presenting with CPE and eligible for NIV were included in the study. After adjusting for age, sex, Charlson Comorbidity Index, smoking status, pH and paO2 using a multivariate Cox regression model, the use of NIV was not associated with a statistically significant reduction in 6-month mortality. However, a notable exception was observed in the subgroup of patients with respiratory acidosis, in whom NIV significantly reduced the risk of death.

These divergent results are consistent with findings in the literature. In 1991, Bersten et al*.* [5] reported no significant mortality benefit from continuous positive airway pressure in patients with CPE and respiratory distress, hypercapnia, and/or acidosis. Similarly, randomized trials by Masip et al. [14] and Nava et al. [15] found no survival advantage in patients with severe CPE treated with CPAP. Notably, these studies included younger populations (mean age of 74 years in Masip et al*.*’s trial) and evaluated a single ventilation mode, unlike the present study, which included bilevel positive airway pressure (BiPAP) ventilation. While several meta-analysis have been published, none has been specifically dedicated to the geriatric population. Pang et al*.*’s 1998 meta-analysis [16], which included few trials and focused solely on CPAP, reported a non-significant trend toward reduced hospital mortality.

Although some studies appear contradictory, recent studies and meta-analysis suggest that an overall survival benefit is associated with NIV use. A prospective study by L’Her et al*.* in 2004 [17], conducted in a population with a similar mean age (84 years), showed improved 48-h in-hospital mortality with NIV and for both ventilation modes. This study also reported a lower average pH (< 7.35), aligning with the results in the subgroup of patients with respiratory acidosis. Masip et al*.*’s 2005 meta-analyise [18], which included 15 trials with a greater emphasis on BiPAP, demonstrated the overall benefit of NIV on mortality in patients admitted for CPE. This effect on mortality was evident for CPAP but not for BiPAP. The 2006 meta-analysis by Winck et al*.* [12], which included 17 randomized controlled trials on NIV's impact on CPE, confirmed these findings, again noting a stronger effect for CPAP than for BiPAP, possibly due to smaller sample sizes in BiPAP trials. Our results suggest that NIV (regardless of ventilation mode) may reduce mortality by over 70% in older patients with CPE and respiratory acidosis (pH < 7.35). This survival benefit, apparent only in patients with a more severe blood gas profile, aligns with population profiles studied in other works that found a reduction in mortality with NIV use. In contrast, the overall cohort may not have had a sufficiently severe clinical profile to demonstrate a statistically significant benefit from NIV [15, 19].

### Secondary outcomes

No significant difference was observed in the length of hospital stay or in the 6-month rehospitalisation rate for CPE. These results are consistent with previous studies that also reported no reduction in hospital stay duration following NIV use in CPE [5, 14]. Evidence from hypercapnic COPD exacerbation studies is more robust, although results remain mixed in older populations. For example, Nava et al. [15] reported similar length of hospital stays in patients over 75 years, while earlier studies in younger patients showed reduced length of stays when NIV was used [20, 21]. This benefit may be linked to the lower use of invasive mechanical ventilation (IMV), a factor that was not clearly evaluable in our study due to the rarity of IMV use.

In the present study, the application of NIV resulted in a greater increase in pH. These findings align with most published data, which have shown more rapid correction of acid–base abnormalities and respiratory rate [4–6]. Unfortunately, the pre- and post-treatment respiratory rate was often undocumented, limiting our ability to assess this parameter.

The prescription rate of NIV was 56%, even though NIV was indicated for each patient based on clinical and gasometric criteria. Respiratory acidosis, hypercapnia and lower peripheral oxygen saturation in ambient air appeared to be predictive factors for the initiation of NIV. Clinical indicators for NIV, such as an elevated respiratory rate and signs of respiratory distress, may have been under-recognised.

Moreover, the significantly higher rate of pulmonary infections in the group receiving standard medical treatment may have influenced clinical decision-making. Pneumonia is a well-known predictor of NIV failure, which may have limited its use in this group.

A review by L’Her et al. questioned the relevance of NIV in patients with respiratory distress due to acute CPE [22], potentially influencing clinical decisions despite official guidelines. This review followed the 2008 multicentre randomised 3CPO trial by Gray et al. [23], which included 1069 patients with respiratory distress (RR > 20/min, pH < 7.35) and clinical features of CPE. The findings contradicted earlier meta-analysis, particularly that of Masip et al. [18], and challenged the 2006 joint SFAR–SRLF consensus. However, methodological limitations of the 3CPO trial raise doubts about its conclusions and do not appear to warrant changes in clinical practice or guideline revisions.

An unexpected finding in our study was the higher prevalence of cognitive impairment and dependency in the NIV + group. These factors did not appear to limit NIV use, although it is possible they were not known at the time of emergency care. Moreover, the depth of cognitive impairment and functional decline was not systematically recorded, limiting interpretation.

### Strengths and limitations of the study

This study specifically focused on frail, polymorbid patients aged 75 years and older, an often underrepresented population in clinical trials. Being a single-center study may limit the generalizability of findings although, as our study population is representative of geriatric patients commonly admitted to emergency departments [24], it provides valuable insights into real-world clinical decision-making in geriatric emergency care.

As the decision to initiate NIV was at the discretion of the emergency physician and not guided by a standardized protocol, despite performing a multivariate Cox regression with adjustments for relevant clinical and gasometric variables, the possibility of residual confounding from unmeasured factors cannot be excluded.

The retrospective study design may also have introduced information bias due to missing clinical data and inconsistent documentation. On the other hand, the reliance on computerized patient records for heart failure coding allowed the recruitment of patients not listed in the emergency book.

Regarding survival data, only in-hospital deaths at the UHS were recorded in the Dx Care^®^ computer file. To minimize loss of survival data, deaths were cross-verified using general practitioner follow-up and the national death registry, ensuring complete data for the primary outcome.

## Conclusion

This retrospective study included 186 frail, older patients with CPE, 56% of whom received noninvasive ventilation (NIV) in accordance with gasometric and clinical criteria. Overall, in this real-life clinical setting, NIV did not significantly reduce 6-month mortality compared to standard treatment. However, in the subgroup of patients with respiratory acidosis (pH < 7.35), NIV was associated with a 76% reduction in the risk of death. These findings suggest that, within a geriatric population, the benefit of NIV in CPE may be limited to patients with acidosis.

Further prospective multicentre studies are warranted to evaluate tolerance, clinical improvement, other key markers of respiratory distress, parametric and ventilatory data, as well as haemodynamic effects and outcomes beyond mere life extension, such as quality of life.

## Data Availability

The datasets used and/or analysed during the current study are available from the corresponding author on reasonable request.
